# From Static to Dynamic: Adaptive Molecular Subtyping in Treated Breast Cancers—Evidence from Single-Center Retrospective Cohort Study

**DOI:** 10.3390/cancers18040657

**Published:** 2026-02-17

**Authors:** Flavia Ultimescu, Carmen Ardeleanu, Octav Ginghina, Mara Mardare, Marius Zamfir, Alina Ioana Puscasu, Irina Bondoc, Andrei-Bogdan Vacarasu, Theodor Antoniu, Ariana Hudita, Bianca Galateanu, Laurentia Gales, Elena Serban, Horia-Dan Liscu, Andreea-Iuliana Ionescu, Mihail Ceausu, Maria-Victoria Olinca

**Affiliations:** 1 OncoTeam Diagnostic S.A., 010719 Bucharest, Romania; flavia.ultimescu@drd.umfcd.ro (F.U.); cmardeleanu@yahoo.com (C.A.); maria.olinca@umfcd.ro (M.-V.O.); 2 Doctoral School of Medicine, University of Medicine and Pharmacy “Carol Davila” Bucharest, 050474 Bucharest, Romania; mara.mardare@drd.umfcd.ro (M.M.); anton-marius-cosmin.zamfir@drd.umfcd.ro (M.Z.); nicoleta-irina.bondoc@drd.umfcd.ro (I.B.); andrei-bogdan.vacarasu@drd.umfcd.ro (A.-B.V.); elena-catalina.serban@drd.umfcd.ro (E.S.); 3 University of Medicine and Pharmacy “Carol Davila” Bucharest, 050474 Bucharest, Romania; octav.ginghina@umfcd.ro (O.G.); mihail.ceausu@umfcd.ro (M.C.); 4 Oncological Surgery 3 Department, “Prof. Dr. A. Trestioreanu” Institute of Oncology Bucharest, 022328 Bucharest, Romania; alina-ioana-puscasu@email.umfiasi.ro (A.I.P.); theodor.antoniu96@gmail.com (T.A.); 5 Doctoral School of Medicine, University of Medicine and Pharmacy “Grigore T. Popa”, 700115 Iași, Romania; 6 Department of Biochemistry and Molecular Biology, University of Bucharest, 050095 Bucharest, Romania; ariana.hudita@bio.unibuc.ro (A.H.); bianca.galateanu@bio.unibuc.ro (B.G.); 7 Oncology Department, University of Medicine and Pharmacy “Carol Davila” Bucharest, 050474 Bucharest, Romania; laurentia.gales@umfcd.ro; 8 Oncology Department, Institute of Oncology “Prof. Dr. Alexandru Trestioreanu”, 022328 Bucharest, Romania; 9 Discipline of Oncological Radiotherapy and Medical Imaging, University of Medicine and Pharmacy “Carol Davila” Bucharest, 020021 Bucharest, Romania; 10 Radiotherapy Department, Colțea Clinical Hospital, 030167 Bucharest, Romania; 11 Medical Oncology Department, Colțea Clinical Hospital, 030167 Bucharest, Romania; 12 Department of Pathology, “C. I. Parhon” National Institute of Endocrinology, 011863 Bucharest, Romania

**Keywords:** breast cancer, longitudinal molecular profiling, adaptive molecular subtyping, treatment resistance, precision oncology, molecular plasticity

## Abstract

**Background/Objective:** Breast cancer (BC) management has traditionally relied on static clinicopathologic and immunohistochemical biomarkers (hormone receptor status, HER2 expression, and proliferative activity assessed at diagnosis). However, these biomarkers are typically evaluated at a single time point and may not reflect therapy-induced molecular evolution. This study evaluates whether longitudinal molecular profiling before and after treatment better characterizes tumor dynamics and provides clinically actionable insights into treatment response, resistance, and prognosis. **Methods:** Thirty-two patients with invasive breast carcinoma were analyzed using histopathology, immunohistochemistry, tissue-based next-generation sequencing, and plasma circulating tumor DNA (ctDNA) analysis. Paired tumor tissue and plasma samples were collected before and after treatment when available. Changes in biomarker expression, molecular subtype, and genomic alterations were assessed to characterize molecular plasticity under therapeutic pressure. **Results:** The cohort had a median age of 54 years (range 29–86), predominantly invasive ductal carcinoma (>85%), and high-grade disease. Hormone receptor-positive tumors accounted for 78.1%. Molecular subtypes were Luminal A (34.4%), Luminal B HER2− (40.6%), Luminal B HER2+ (6.3%), HER2-enriched (6.3%), and triple-negative breast cancer (12.5%). Initial tissue sequencing identified PI3K/AKT pathway alterations in 28.1% of cases. Post-treatment analyses revealed substantial molecular discordance, including progesterone receptor loss (33.3%), HER2 status changes (33.3%), and Ki67 variability (77.8%). Plasma ctDNA analysis was informative in 53.1% of patients and identified additional clinically relevant alterations, including FGFR1 amplification and BRCA1/2 variants not detected in tissue. **Conclusions:** BC molecular profiles are dynamic and frequently altered by therapy. Longitudinal molecular assessment reveals clinically actionable changes overlooked by static subtyping, supporting a dynamic model of molecular classification, highlighting the potential value of adaptive molecular subtyping to improve treatment stratification and resistance monitoring.

## Simple Summary

Breast cancer treatment decisions are commonly based on tumor characteristics assessed at the time of diagnosis. However, breast cancer is a biologically dynamic disease, and tumor molecular profiles can change substantially under therapeutic pressure. Relying on a single baseline evaluation may therefore fail to capture clinically relevant biological evolution that influences treatment response and resistance. In this study, we investigated treatment-associated molecular dynamics by integrating paired tissue-based and blood-based analyses obtained before and after therapy. We observed frequent changes in hormone receptor expression, proliferative activity, HER2 status, and genomic alterations, highlighting substantial molecular plasticity during treatment. Circulating tumor DNA analysis provided complementary information, revealing additional alterations not captured by tissue sampling alone and reflecting tumor heterogeneity across disease compartments. Rather than proposing a new molecular classification system, this study provides biological and translational evidence for dynamically reassessing breast cancer molecular states to enable more personalized, adaptive treatment strategies.

## 1. Introduction

Breast cancer is a biologically heterogeneous disease, and molecular subtyping has played a central role in guiding its clinical management [1]. The conventional intrinsic subtypes—Luminal A, Luminal B, HER2-enriched, and triple-negative—originating from gene expression profiling and immunohistochemical surrogates, continue to be essential for therapeutic decision-making [2]. In standard clinical practice, these subtypes are typically assigned at the time of diagnosis based on a single tumor biopsy, offering merely a limited view of a biologically dynamic process. Increasing evidence suggests that molecular subtypes assigned at initial diagnosis incompletely reflect intratumoral heterogeneity and do not adequately capture therapy-induced molecular remodeling, which can substantially influence clinical outcomes.

Accumulating genomic and transcriptomic data demonstrate that breast cancer evolves under selective pressure imposed by systemic therapies, including endocrine therapy, chemotherapy, and targeted agents [3]. Real-world observational data from patients treated with CDK4/6 inhibitors demonstrate heterogeneous clinical outcomes that likely reflect underlying biological diversity and therapy-driven tumor evolution not captured by initial molecular classification alone at diagnosis [4]. Treatment exposure drives clonal selection, epigenetic reprogramming [5], receptor conversion, and the acquisition of resistance-associated genomic alterations such as *PIK3CA*, *ESR1*, *TP53*, and DNA damage–repair pathway mutations. Longitudinal analyses of paired pre- and post-treatment tumor samples have documented frequent changes in hormone receptor status [6], HER2 expression, and Ki67 proliferative index [7], challenging the assumption that molecular subtype at diagnosis reliably predicts long-term tumor behavior. Spatial heterogeneity further limits the representativeness of single core biopsy specimens, particularly in locally advanced and metastatic disease [8].

Neoadjuvant chemotherapy (NACT) provides the clinical framework for studying therapy-induced molecular evolution, enabling systematic comparison of tumor biology before and after treatment.

Recent evidence indicates that shifts in molecular markers, such as hormone receptors, proliferation index, and intrinsic subtype, following neoadjuvant therapy are not only common but also carry independent prognostic significance, with changes in these profiles associated with outcomes, including survival and recurrence, beyond what baseline classification predicts. These dynamic alterations suggest that relying solely on static subtyping at diagnosis may overlook tumors that become biologically less aggressive with treatment and may overestimate risk in patients who demonstrate a favorable molecular response [9].

In this context, adaptive molecular subtyping has emerged as a complementary paradigm to classical intrinsic classification. Instead of relying solely on static baseline gene expression profiles, adaptive subtyping incorporates therapy-induced transcriptional changes to capture how tumors biologically evolve under treatment pressure [10]. Translational analyses embedded within randomized neoadjuvant clinical trials have demonstrated that adaptive molecular clusters stratify patients by prognosis more accurately than intrinsic subtypes alone, identifying subsets with excellent long-term outcomes despite residual disease. In the Penelope-B trial [11], Denkert et al. analyzed longitudinal transcriptomic profiles from paired pre- and post-neoadjuvant chemotherapy samples and used 335 differentially expressed genes to derive five therapy-response-linked adaptive clusters (AC-1 to AC-5). These adaptive clusters identified a large low-risk group with excellent prognosis (AC-1 and AC-2), intermediate-risk groups (AC-3 and AC-4), and a very poor-prognosis group enriched for basal-like/HER2-enriched biology (AC-5). Adaptive subtyping also revealed pronounced shifts in intrinsic subtype, including transitions from luminal B to luminal A after neoadjuvant therapy and reverse transitions in metastatic samples, emphasizing that intrinsic subtypes are dynamic under chemotherapy. Thus, paired-sample-informed adaptive subtyping can more precisely identify high-risk subpopulations among clinically resistant tumors and provides a biologically rational basis for refined post-neoadjuvant risk stratification and therapy development.

**Figure 1:**
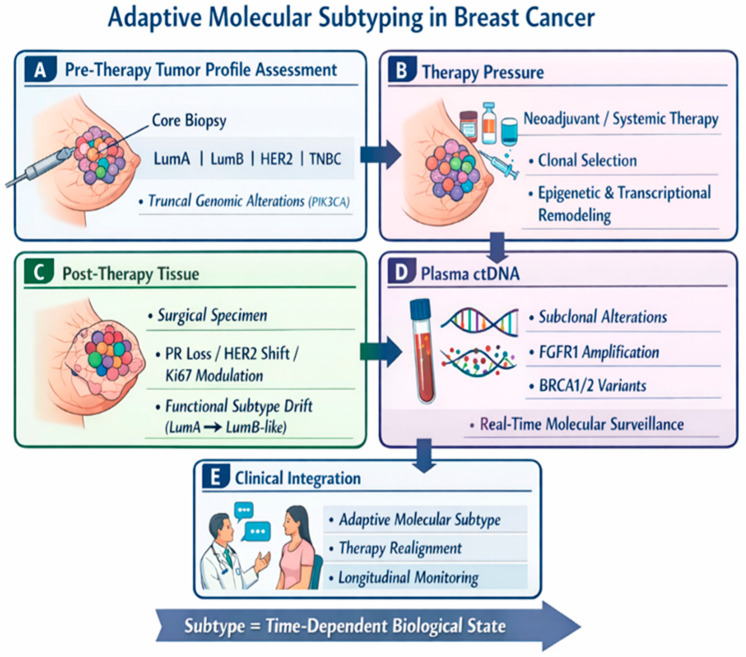
Adaptive molecular subtyping in breast cancer. This schematic depicts molecular subtypes as dynamic, time-dependent states shaped by therapy. (**A**) Pre-therapy tumor profile assessment: Core biopsy defines intrinsic subtype and truncal genomic alterations. (**B**) Therapy pressure: Systemic treatment drives clonal selection and transcriptional/epigenetic remodeling. (**C**) Post-therapy tissue: Surgical specimens show biomarker changes and functional subtype drift. (**D**) Plasma ctDNA: Liquid biopsy detects emerging subclonal alterations in real time. (**E**) Clinical integration: Longitudinal data inform adaptive subtyping, therapy realignment, and monitoring.

Complementing tissue-based profiling, liquid biopsy—particularly circulating tumor DNA (ctDNA) analysis—has emerged as a minimally invasive approach to capture real-time tumor heterogeneity and molecular evolution. ctDNA reflects the composite genomic landscape of primary and metastatic lesions and enables longitudinal monitoring of clonal dynamics, minimal residual disease, and emergent resistance alterations that are frequently undetectable in tissue biopsies. Recent prospective studies in early-stage and metastatic BC have shown that ctDNA detection often precedes clinical or radiographic relapse by several months and provides prognostic information independent of standard clinical and pathological features across subtypes [12,13]. These findings support its integration into adaptive treatment strategies, where serial ctDNA assessment may guide risk stratification and early therapeutic intervention (Figure 1).

Emerging evidence indicates that molecular subtype transitions in breast cancer occur not only as a consequence of therapy but also during natural disease progression, such as metastatic setting. Notably, reverse subtype transitions have been described, in which tumors shift from more aggressive phenotypes—such as basal-like or luminal B—toward less aggressive subtypes, including luminal A or normal-like, in metastatic lesions [14,15]. This phenotypic plasticity likely reflects adaptation to distinct microenvironmental pressures, therapy-independent clonal selection, or the expansion of subclonal populations with divergent transcriptional programs [16]. Importantly, such subtype shifts carry meaningful prognostic and therapeutic implications, as the molecular characteristics of metastatic disease may differ substantially from those of the primary tumor and directly influence sensitivity to systemic therapies. Consequently, re-biopsy and molecular recharacterization of metastatic sites are increasingly recognized as integral components of personalized treatment strategies in advanced breast cancer [17].

In this study, we investigate this dynamic disease model by integrating paired tissue- and plasma-based genomic profiling obtained before and after therapy to characterize molecular subtype plasticity and to evaluate its relevance for longitudinal disease assessment. Therapy-associated phenotypic drift and subtype discordance in breast cancer have been widely reported, including receptor conversion and transitions toward more aggressive phenotypes. Therefore, the novelty of the present study is not the observation that subtype switching occurs, but the integrated longitudinal, multi-compartment assessment of treated breast cancers using paired diagnostic core biopsy and post-treatment surgical (mastectomy) specimens together with plasma ctDNA profiling. This approach enables simultaneous evaluation of therapy-associated remodeling in clinically used subtype-defining markers (PR, HER2 score, and Ki67) alongside genomic alterations, including events detectable only in plasma.

## 2. Materials and Methods

### 2.1. Study Design and Patient Cohort

This observational study comprised 32 breast cancer cases (female patients aged 29 to 86 years) with paired immunophenotypic and molecular data collected between 2023 and 2024 in the Department of Surgery at Memorial Hospital in Bucharest, Romania. This study was approved as part of the main research project ONCOGUARD PN-III-P2-2.1-PTE-2021-0663. Patients signed specific informed consent to be recorded and included in the qualitative analysis. Because treatment regimens and outcome endpoints were not uniformly available, this study is hypothesis-generating and does not test associations with survival or response. The study is limited by its single-center, retrospective design, which introduces potential selection bias and limits generalizability. Paired surgical samples were not available for all patients, and the evaluable subset may not fully represent the entire cohort.

Data collection. Clinicopathological and molecular variables were extracted retrospectively from pathology and molecular reports when available. Collected variables included patient age, histopathological diagnosis, and histological grade, hormone receptor (HR) and HER2 status, Ki67 proliferation index, molecular subtype, and genomic alterations identified by next-generation sequencing (NGS).

Specimen types. Samples analyzed were classified into three categories:

1. Core biopsy specimens, representing diagnostic tissue obtained at initial presentation, were used for histopathological evaluation, immunohistochemistry (IHC), molecular subtype assignment, and tissue-based NGS when available.
2. Plasma samples were analyzed using circulating tumor DNA (ctDNA)/cell-free DNA (cfDNA) NGS assays.
3. Surgical specimens (mastectomy samples), when available, were subjected to histopathological examination and immunophenotypic and molecular analyses to assess post-therapy changes.

Pre-analytical tissue handling and fixation procedures were performed in accordance with established histopathological preservation standards to ensure antigen and nucleic acid integrity [18].

### 2.2. Longitudinal Biomarker Change—For Paired Baseline–Surgical Comparisons, the Following Definitions Were Applied

- Receptor conversion: change in ER and/or PR status between core biopsy and surgical specimen;
- HER2 score variability: any change in HER2 IHC score between time points;
- Ki67 variation: absolute change in Ki67 expressed as percentage points

Surgical specimens were available in 18 of 32 cases (56.3%). Plasma ctDNA results were considered informative when a mutation result was reported other than “Invalid” or missing/NA. Based on this definition, plasma results were informative in 17/32 cases (53.1%), invalid in 8/32 cases (25.0%), and missing or not available in 7/32 cases (21.9%).

Histological evaluation was performed on core biopsy specimens and mastectomy samples after immersion in 10% neutral buffered formalin, fixation, and subsequent automatic processing and paraffin embedding.

Immunohistochemistry and biomarker assessment. Immunohistochemical (IHC) analysis was carried out using the Ventana BenchMark Ultra system (Ventana Medical Systems, 1910 E. Innovation Park Drive, Tucson, AZ 85755, USA). Estrogen receptor (ER) and progesterone receptor (PR) expression were classified as positive or negative, with the exact percentage of positive tumor cells documented. HER2 status was assessed by immunohistochemistry (IHC) and reported using ASCO/CAP standard scoring criteria (0, 1+, 2+, or 3+) [19]. For equivocal HER2 expression (IHC 2+), silver in situ hybridization (SISH) results were recorded and categorized as amplified or nonamplified. The Ki67 proliferation index was recorded as a percentage of positively stained tumor cells.

Pre-treatment molecular subtype was recorded using standard clinical classifications: Luminal A, Luminal B, Luminal B HER2-positive, HER2-enriched, and triple-negative breast cancer (TNBC), according to the St Gallen classification [20].

All 32 diagnostic core biopsy specimens were subjected to tissue-based next-generation sequencing (NGS) using the Oncomine Comprehensive Assay Plus panel (Ion Torrent platform, 168 Third Avenue, Waltham, MA 02451, USA). Tissue NGS was therefore performed uniformly across the entire cohort, ensuring complete genomic coverage of baseline biopsy samples for downstream molecular analyses. TP53 status was determined exclusively by NGS, and p53 immunohistochemistry was not performed as a surrogate for mutation status.

Limitations of Longitudinal Biomarker ChangeA limitation of longitudinal subtype assessment is distinguishing true biological evolution from technical or sampling-related variability. Discordance between baseline core biopsies and post-treatment surgical specimens may arise from intratumoral spatial heterogeneity, differences in tumor cellularity and stromal admixture, or sampling of biologically distinct regions, particularly after therapy-induced remodeling. In addition, pre-analytical factors (e.g., fixation and tissue processing) and assay-level variability (including interobserver variation in IHC scoring and sensitivity thresholds in NGS) can influence biomarker quantification and downstream subtype classification. Although tissue handling followed established histopathology preservation standards and biomarker assessment was performed using standardized clinical workflows, this retrospective cohort lacked systematic tumor purity estimates, replicate testing, and internal reproducibility metrics across timepoints, limiting our ability to fully exclude methodological drift as a contributor to apparent subtype switching. Nevertheless, the observed directionality and patterns of change—such as selective PR loss in ER-positive disease, HER2 score variability, and dynamic Ki67 remodeling under treatment pressure—are biologically plausible and consistent with therapy-associated molecular plasticity reported in larger paired-sample studies.

### 2.3. Tissue NGS and Plasma ctDNA Analysis

Molecular analysis using next-generation sequencing (NGS) was performed on formalin-fixed, paraffin-embedded biopsy samples and RNA and DNA were extracted from solid tumor tissue, while circulating tumor DNA (ctDNA) and a fraction of circulating free DNA (cfDNA) were analyzed from plasma samples. Sequencing was performed using the Ion Torrent platform (Thermo Fisher Scientific) with the Oncomine Comprehensive Assay Plus (OCA Plus), which interrogated approximately 500 cancer-related genes. The analysis revealed a range of genomic alterations, including point mutations, single-nucleotide variants (SNVs), insertions and deletions (indels), copy number variations (CNVs), and gene fusions.

Variants were classified according to the terminology used in the dataset as pathogenic variants (PV), variants of uncertain significance (VUS), or unclassified variants (UPV). Copy-number alterations were recorded when explicitly reported.

### 2.4. Statistical Analysis

Primary and secondary endpoints. To reduce multiplicity and improve interpretability, analyses were structured hierarchically. Primary paired endpoints in the biopsy–surgical subset were predefined as: (i) progesterone receptor (PR) conversion rate, (ii) HER2 IHC score discordance rate (0–3+), and (iii) paired Ki67 change (ΔKi67 = surgical − baseline). All remaining analyses (including ER conversion, subtype transitions, ctDNA feasibility metrics, and gene-level alteration frequencies) were treated as secondary/exploratory.

Hypothesis testing framework. For paired binary biomarkers (ER and PR), discordance was tested using McNemar’s exact test, corresponding to the null hypothesis of symmetric conversion (loss and gain equally likely). Agreement beyond chance was quantified using Cohen’s κ. For HER2 IHC score treated as an ordinal categorical variable (0, 1+, 2+, 3+), agreement was quantified using weighted Cohen’s κ with quadratic weights, and score-level shifts were evaluated using an ordinal paired symmetry test (Bowker’s test of symmetry or Stuart–Maxwell test), corresponding to the null hypothesis of marginal homogeneity across timepoints. Ki67 was analyzed as a paired continuous variable using the Wilcoxon signed-rank test, with effect size estimated using rank-biserial correlation.

Multiple comparisons. Given the exploratory nature and modest sample size, formal inference was restricted to the predefined primary endpoints. For secondary hypothesis tests, false discovery rate (FDR) control was applied using the Benjamini–Hochberg procedure, and both nominal *p*-values and FDR-adjusted q-values are reported when applicable. Statistical significance was interpreted cautiously, with emphasis placed on effect sizes, confidence intervals, agreement metrics, and biological plausibility rather than binary significance thresholds.

Subgroup analysis policy. Subgroup analyses (e.g., subtype-stratified discordance patterns) were prespecified as exploratory and were not subjected to formal hypothesis testing when any subgroup cell count was <5. Such results are presented descriptively, with exact confidence intervals to reflect the imprecision due to small denominators.

Sensitivity analyses. To assess the robustness of discordance estimates, sensitivity analyses were performed focusing on clinically meaningful categorization and measurement variability. Specifically, HER2 discordance was additionally summarized using boundary-crossing categories (e.g., HER2-negative vs. HER2-low vs. HER2-positive, where applicable) rather than raw score shifts alone. Ki67 change was evaluated both continuously (ΔKi67) and categorically using clinically meaningful thresholds (e.g., ≥10 percentage-point decrease vs. other), to reduce susceptibility to scoring variability.

Categorical variables are summarized as counts and percentages with corresponding 95% confidence intervals (Wilson method). Continuous variables are reported as median and interquartile range (IQR). Paired analyses were performed for cases with both diagnostic core biopsy and surgical specimens available. Agreement between paired categorical biomarkers (estrogen receptor [ER] and progesterone receptor [PR]) was assessed using Cohen’s kappa coefficient (κ). Paired discordance for binary receptor status was evaluated using McNemar’s exact test. For HER2 immunohistochemistry (IHC) scores, treated as an ordinal variable (0, 1+, 2+, 3+), agreement between paired specimens was assessed using weighted Cohen’s kappa with quadratic weights; HER2 score changes were additionally summarized by directionality (upward vs. downward shifts) and by clinically relevant boundary-crossing transitions. Ki67 proliferation index was analyzed as a paired continuous variable. Ki67 values are summarized as median (IQR), and within-patient changes (ΔKi67 = surgical − baseline) were evaluated using the Wilcoxon signed-rank test. The effect size for the paired change in Ki67 was estimated using the rank-biserial correlation.

Molecular subtype evolution between baseline and surgical specimens was summarized descriptively using paired transition frequencies. The feasibility of plasma circulating tumor DNA (ctDNA) assays was summarized descriptively as informative, invalid, or unavailable, and results were interpreted in the context of tumor phenotype.

All analyses were exploratory and hypothesis-generating. Given the limited sample size and incomplete longitudinal sampling, statistical significance was interpreted cautiously, with emphasis placed on effect sizes, agreement metrics, and biological plausibility rather than formal hypothesis testing.

### 2.5. Study Limitations

This study is exploratory and limited by its retrospective design, modest sample size, and incomplete longitudinal sampling. Treatment regimens were heterogeneous, and survival outcomes were not uniformly available, precluding definitive prognostic conclusions. Consequently, this study cannot determine whether subtype switching directly alters clinical management, predicts outcomes, or improves prognostic stratification beyond baseline classification.

In addition, ctDNA interpretation was constrained by assay validity and challenges in variant classification.

Nonetheless, the consistency of observed molecular drift patterns with prior large-scale studies supports the biological plausibility of our findings.

Due to modest sample size and incomplete longitudinal sampling, this study was not powered for definitive subgroup inference or multivariable modeling. Accordingly, subgroup analyses were treated as exploratory and interpreted primarily in terms of effect sizes and confidence intervals rather than *p*-values. While we implemented a hierarchical endpoint structure and FDR correction for secondary analyses, the results should be considered hypothesis-generating and require validation in larger prospective cohorts with standardized sampling and treatment stratification.

## 3. Results

### 3.1. Pre-Treatment Molecular and Immunophenotypic Profile

#### 3.1.1. Histopathology Assessment (Histological Subtype and Grade)

Histopathological evaluation of pre-treatment core biopsy specimens (*n* = 32) demonstrated a marked predominance of invasive ductal carcinoma (IDC), which accounted for 30 of 32 cases (93.8%). Invasive lobular carcinoma (ILC) accounted for a minority of cases in the analyzed cohort, with 2 cases (6.2%). Within IDC, high-grade tumors (Nottingham grade 3) were overrepresented. Overall, 16 of 32 cases (50.0%) were classified as grade 3, while 12 cases (37.5%) were grade 2, and 4 cases (12.5%) were grade 1. This distribution indicates a substantial burden of biologically aggressive disease already present at diagnosis.

Patients presenting with lymph node metastases at diagnosis (6/32, 18.8%) demonstrated clinicopathological features consistent with a more aggressive biological phenotype compared with primary tumor biopsies. High histological grade predominated in nodal metastases, with 5 of 6 cases (83.3%) classified as Nottingham grade 3, supporting an association between nodal involvement and poor differentiation. Proliferative activity was also increased in metastatic lymph node samples, with a median Ki67 index of 42.5% (range, 25–90%), compared with a median Ki67 of 30% in primary tumors. From a molecular standpoint, nodal disease was associated with a higher prevalence of aggressive subtypes, with luminal B tumors comprising 66.7% (4/6) of cases and triple-negative breast cancer accounting for 33.3% (2/6), proportions higher than those observed in the overall cohort. Together, these observations indicate that biologically aggressive features are frequently present at initial diagnosis in patients with lymph node metastases.

#### 3.1.2. Hormone Receptor Expression Pre-Treatment

On core-biopsy immunohistochemistry (n = 32), ER positivity was observed in 25/32 cases (78.1%) and PR positivity in 24/32 cases (75.0%), consistent with a high prevalence of hormone receptor (HR)-positive disease. Combined HR categories were distributed as ER+/PR+ in 24/32 (75.0%), ER+/PR− in 1/32 (3.1%), and ER−/PR− in 7/32 (21.9%); ER−/PR+ was not observed (0/32; 0.0%). Although uncommon in this cohort (1/25 ER-positive tumors; 4.0%), the ER+/PR− phenotype is widely recognized as a clinically relevant discordant subgroup. Evidence from clinical and translational studies supports that ER+/PR− tumors, compared with ER+/PR+ disease, are more frequently associated with inferior endocrine responsiveness and less favorable outcomes, particularly within higher-risk luminal groups. Although ER signaling remains active, PR loss—caused by genetic alterations (copy-number loss), epigenetic silencing (promoter/exon methylation), miRNA regulation, growth factor pathway activation (notably PI3K/AKT/mTOR and HER-2), or treatment-induced changes—disrupts normal hormone responsiveness. Clinically, ER+PR− tumors occur more frequently in older and postmenopausal women, display higher proliferation indices, larger tumor size, and more aggressive pathological features, with survival outcomes approaching those of ER−PR− tumors. Genomically, they exhibit greater instability and characteristic alterations such as TP53 mutations and ZNF703/RPS6KB1 amplifications. Therapeutically, ER+PR− breast cancer shows reduced benefit from tamoxifen and a higher likelihood of early endocrine resistance, highlighting the need for tailored strategies that incorporate pathway-targeted and potentially epigenetic therapies [21].

Formal testing of discordance patterns. In the paired biopsy–surgical subset (n = 18), ER status remained fully concordant (0/18 discordant), indicating no ER gain or loss in this cohort. PR discordance was observed in 6/18 cases (33.3%) and was strictly directional (loss only), rejecting the null hypothesis of symmetric conversion (McNemar exact *p* = 0.031). HER2 IHC score discordance occurred in 6/18 cases (33.3%), with both upward and downward score shifts observed. Using Bowker’s symmetry test applied to the paired 4 × 4 HER2 score table, HER2 score transitions were consistent with symmetric discordance rather than a systematic directional shift (*p* = 0.57), supporting bidirectional variability across sampling timepoints. Ki67 showed frequent within-patient remodeling (14/18; 77.8%), with a predominance of decreases, consistent with a treatment-associated suppression signal in many tumors (Table 1). 

#### 3.1.3. Pre-Treatment Ki67

Demonstrated substantial intertumoral heterogeneity in this cohort (n = 32) and was integral to baseline subtype allocation. Ki67 distributions were subtype-stratified: Luminal A tumors (n = 11) showed low proliferative activity (median 12%, IQR 10–14.5%, range 5–18%), whereas Luminal B tumors (n = 13) had higher proliferation (median 30%, IQR 30–45%, range 25–60%). TNBC (n = 4) exhibited the highest Ki67 values (median 70%, IQR 55–82.5%, range 40–90%). HER2-enriched tumors (n = 2) showed marked variability (range 12–80%, median 46%), consistent with biological heterogeneity in this small subgroup.

Biologically, Ki67 is a quantitative measure of proliferative capacity, closely associated with aggressive clinicopathologic features and potentially reflective of replicative stress and genomic instability. Beyond its role in baseline subtype discrimination (Luminal A-like versus Luminal B-like), accumulating evidence indicates that dynamic changes in Ki67 during neoadjuvant therapy carry prognostic and predictive significance, supporting its utility in capturing evolving tumor biology under therapeutic selection pressure [22].

From a methodological standpoint, contemporary expert consensus continues to support Ki67 as a clinically informative biomarker, particularly for refining luminal risk stratification, provided that standardized pre-analytical conditions and reproducible scoring methodologies are applied [23]. Nevertheless, persistent inter-observer and inter-laboratory variability remains well recognized, especially within intermediate Ki67 ranges, underlining the need for cautious interpretation. Recent real-world, multi-institutional data support the adoption of International Ki67 Working Group-aligned global scoring approaches, which improve concordance with hotspot-based assessments [24].

### 3.2. Pre-Treatment Genomic Alterations

#### 3.2.1. PIK3CA Pathogenic Variants

PIK3CA pathogenic variants activate the PI3K–AKT–mTOR signaling axis, promoting tumor cell proliferation and survival while reducing dependence on estrogen receptor (ER)-mediated transcription. Dysregulation of this pathway is repeatedly implicated in mechanisms of endocrine resistance in hormone receptor (HR)-positive breast cancer [25].

In our study, PIK3CA PVs were identified in 7 of 32 cases (21.9%) by next-generation sequencing of core biopsy specimens. These alterations were predominantly detected in ER-positive tumors, occurring in 6 of 25 (24.0%). PIK3CA PVs were absent in ER-negative tumors, except for a single HER2-enriched case (ER−/PR−/HER2 3+). Subtype-stratified analysis demonstrated a non-uniform distribution: PIK3CA PVs were present in 4 of 11 Luminal A tumors (36.4%) and 2 of 13 Luminal B tumors (15.4%), while no variants were detected in Luminal B HER2+ (0/2) or triple-negative breast cancer (0/4) cases. Despite a lower overall frequency compared with large genomic datasets, the preferential occurrence in luminal tumors and absence in triple-negative disease align with prior large-scale genomic observations [25].

The clinical relevance of PIK3CA alterations is well established, with guideline-endorsed targeted therapies such as PI3Kα inhibition (e.g., alpelisib combined with fulvestrant) and broader pathway-directed strategies for tumors harboring PIK3CA, AKT1, or PTEN alterations [26]. Within the present cohort, the baseline enrichment of PIK3CA PVs in luminal tumors (particularly Luminal A) supports interpretation of these cases as having pre-existing activation of PI3K-pathway signaling, a molecular context that may be associated with attenuated endocrine sensitivity [27] and potential suitability for pathway-targeted combinations when clinically indicated.

#### 3.2.2. AKT and Downstream Signaling Alterations

As outlined above, activation of the PI3K–AKT–mTOR signaling axis promotes tumor cell survival and growth and can reduce functional dependence on estrogen receptor (ER) mediated transcription, thereby contributing to endocrine resistance in hormone receptor (HR) positive BC [26]. In the pre-treatment setting, AKT pathogenic variants (PVs) were identified in 2 of 32 cases (6.3%). Both AKT-altered tumors were HR-positive (2/2; 100% ER+/PR+) and occurred exclusively within luminal disease, including 1 of 11 Luminal A cases (9.1%) and 1 of 13 Luminal B cases (7.7%). No AKT PVs were detected in Luminal B HER2+ (0/2), HER2-enriched (0/2), or triple-negative breast cancer (0/4). The two AKT-altered tumors exhibited intermediate proliferative activity within the luminal spectrum (Ki67 18% and 25%, respectively), with low HER2 expression (IHC 1+ in both cases).

This distribution suggests that AKT pathway alterations may arise prior to the development of overt high-proliferation phenotypes, potentially representing an early adaptive signaling state within ER-driven tumors. Such alterations may precede more aggressive clinicopathologic features and contribute to the development of endocrine resistance.

Recent clinical evidence and expert consensus support therapeutic targeting of AKT-pathway-altered tumors in advanced HR-positive/HER2-negative breast cancer. In the CAPItello-291 trial, the addition of the AKT inhibitor capivasertib to fulvestrant significantly improved clinical outcomes, with biomarker-enriched benefit observed in tumors harboring PIK3CA, AKT1, or PTEN alterations. Recent clinical evidence and expert consensus support therapeutic targeting of AKT-pathway–altered tumors in advanced HR-positive/HER2-negative breast cancer. In the CAPItello-291 trial, the addition of the AKT inhibitor capivasertib to fulvestrant significantly improved clinical outcomes, with biomarker-enriched benefit observed in tumors harboring PIK3CA, AKT1, or PTEN alterations [26]. Although the present cohort includes only two baseline AKT PV cases, their restriction to luminal disease is biologically coherent and supports consideration of PI3K/AKT pathway evaluation as part of risk-adapted treatment planning in ER-positive tumors, particularly when accompanied by other indicators of aggressive potential, such as rising Ki67, progesterone receptor loss, or ctDNA evidence of molecular evolution [26].

#### 3.2.3. TP53, BRCA1/2, and MYC Alterations

Alterations associated with genomic instability and aggressive biology were uncommon overall but showed subtype-specific patterns in this cohort.

TP53. A TP53 pathogenic variant (PV) was detected in 1/32 cases (3.1%) and was confined to the HER2-enriched subtype (1/2; 50.0%), with no TP53 PV identified at baseline in luminal tumors (0/24) or TNBC (0/4). This distribution is biologically plausible, as TP53 alterations are subtype-associated and are frequently linked to adverse outcomes in prior studies [28].

BRCA1/2. No BRCA1/2 PVs were identified in baseline tissue NGS (0/32). However, plasma ctDNA detected BRCA1 and BRCA2 variants (reported as UPV) in a TNBC case, indicating that clinically relevant DNA-repair alterations may be missed by localized tissue sampling or emerge in a different compartment/timepoint. This is consistent with guidance supporting reassessment of disease biology during progression and with the therapeutic relevance of PARP inhibition in BRCA1/2-mutated HER2-negative disease [29].

MYC. MYC alterations were infrequent in core biopsy tissue (MYC amplification, 1/32 [3.1%]; MYC VUS, 1/32 [3.1%]; total 2/32 [6.3%]). The MYC VUS occurred in a case presenting with lymph node metastasis at diagnosis, suggesting that MYC-associated signals may be enriched in biologically advanced presentations even when rare at baseline. MYC is widely recognized as a driver of proliferation, metabolic reprogramming, and therapy resistance across cancers, including breast cancer, and remains an active area of therapeutic investigation [30].

Even at low frequency, identification of TP53, BRCA1/2, and MYC alterations delineates a subgroup with biology consistent with genomic instability and potential clonal diversification, supporting intensified molecular surveillance and, where appropriate, implementation of DNA-damagedirected strategies (e.g., PARP inhibitors in BRCA-altered HER2-negative disease) and consideration of clinical trial enrollment for MYC- or instability-associated approaches.

### 3.3. Plasma ctDNA Analysis

Plasma circulating tumor DNA (ctDNA) profiling was integrated to complement tissue-based genomic assessment and to capture tumor heterogeneity. Unlike tissue sequencing, which reflects a single sampled lesion at a single time point, ctDNA provides a composite signal reflecting contribution from multiple tumor compartments, including occult metastatic deposits, and can therefore serve as a dynamic biomarker of tumor burden and clonal composition. In this cohort, plasma testing generated informative ctDNA results in 17/32 cases (53.1%), while 8/32 (25.0%) were reported as invalid, and 7/32 (21.9%) were not available. Informative plasma results were observed across molecular subtypes, including Luminal B (8/13; 61.5%), Luminal A (5/11; 45.5%), TNBC (3/4; 75.0%), and HER2-enriched (1/2; 50.0%), whereas Luminal B HER2+ (0/2; 0.0%) had no informative plasma result recorded.

#### 3.3.1. Genomic Alterations Detected Exclusively in Plasma Samples

Although the majority of informative plasma results were reported as variants of uncertain pathogenicity (UPV) and only rarely as variants of uncertain significance (VUS), circulating tumor DNA (ctDNA) analysis provided clinically relevant information in selected cases. Specifically, plasma testing identified molecular alterations that were not captured by pre-treatment tissue analysis, either because tissue profiling was negative or tissue material was unavailable. In this dataset, actionable or biologically meaningful plasma-only findings not reported in baseline tissue were observed in 2 of 32 cases (6.3%).

FGFR1 amplification was detected in plasma in 1 of 32 cases (3.1%), occurring in a TNBC case in which tissue next-generation sequencing was reported as pathogenic variant-negative. In addition, BRCA1 and BRCA2 alterations were identified in plasma as UPV in 1 of 32 cases (3.1%), also in a TNBC case in which pre-treatment tissue molecular evaluation was unavailable.

Although the absolute frequency of such events was low, these observations provide cohort-level evidence that ctDNA analysis can (i) detect spatially distinct tumor clones not represented in a single biopsy specimen and/or (ii) recover clinically actionable genomic information when tissue profiling is non-informative or not feasible. The identification of FGFR1 amplification and BRCA1/2 alterations solely in plasma is consistent with the well-established capacity of ctDNA to detect clinically relevant genomic changes that may be missed by tissue-based analyses due to sampling limitations, tumor heterogeneity, or limited tissue availability.

Notably, FGFR pathway activation has strong biological plausibility in breast cancer progression and treatment resistance. Its detection in TNBC plasma in this cohort reinforces the complementary value of plasma-based testing in uncovering molecular alterations beyond baseline tissue phenotype and highlights the potential role of ctDNA in longitudinal disease monitoring.

#### 3.3.2. ctDNA Interpretive Constraints: Variant Classification and Assay Validity

Plasma results in this cohort also highlight common implementation challenges in routine care. Non-definitive classifications were frequent: UPV was reported in 13/32 cases (40.6%), and VUS in 1/32 (3.1%); among informative plasma tests, this corresponded to UPV in 13/17 (76.5%) and VUS in 1/17 (5.9%). Among informative plasma tests, this corresponds to UPV in 13/17 (76.5%) and VUS in 1/17 (5.9%). UPV calls were most common in Luminal B (7/13; 53.8%) and Luminal A (4/11; 36.4%), with fewer in TNBC (1/4; 25.0%), while the single VUS call (TP53 VUS) occurred in TNBC.

Contemporary reviews and consensus discussions similarly emphasize that ctDNA utility depends on analytical validity, consistent reporting, and careful integration into clinical decision-making rather than isolated interpretation of borderline calls. The European Liquid Biopsy Society Workshop consensus emphasizes that ctDNA results must be interpreted by integrating variant confidence (analytical validity and classification), the clinical context (tumor type, stage, burden, treatment status), and corroborating tissue/phenotypic data when available. The guidance cautions against isolated interpretation of low-confidence or borderline calls, recommending transparent reporting (e.g., LoB/LoD, equivocal labeling), clinical disclaimers, and integration into multidisciplinary decision-making to ensure appropriate clinical use [31].

Within this setting, ctDNA functions as a real-time molecular sentinel: it can flag emergent pathways (e.g., FGFR amplification), reveal DNA-repair alterations when tissue is unavailable, and provide additional molecular context before (or even without) clear phenotypic conversion in tissue. This integrated approach aligns with modern precision oncology practice, where serial molecular profiling can guide adaptation of treatment strategies over time rather than relying solely on baseline subtype labels. While plasma was not universally informative and many results were non-definitive (UPV/VUS), the cohort demonstrates that ctDNA can contribute non-redundant, clinically relevant information in selected patients. Even a small number of plasma-only actionable events (6.3%) is meaningful in a framework that aims to intercept resistance early, because these events represent situations where tissue-only strategies would have missed or delayed identification of targetable biology.

### 3.4. Phenotypic/Genomic Drift Between Core Biopsy and Resection Specimens: Evidence of Molecular Drift/Plasticity and Implications for Resistance/Therapy Response

Paired cohort definition and null-hypothesis testing. Paired baseline–surgical biomarker comparisons were available in 18/32 cases (56.3%) based on the presence of both diagnostic core biopsy and mastectomy specimen data.

Within this paired cohort (n = 18), ER status remained stable (0/18 conversions), supporting high longitudinal concordance of ER positivity/negativity across sampling time points in this dataset.

In contrast, PR conversion occurred in 6/18 cases (33.3%) and was strictly directional (PR loss only, with no PR gain events), rejecting the null hypothesis of symmetric conversion (McNemar exact *p* = 0.031).

HER2 IHC score discordance (0–3+) was observed in 6/18 paired cases (33.3%), reflecting measurable score-level variability between baseline biopsy and surgical specimen.

To formally test whether HER2 transitions represented systematic directional drift rather than symmetric disagreement, Bowker’s symmetry test was applied to the paired 4 × 4 HER2 score table and showed no evidence of directional redistribution (*p* = 0.57), consistent with bidirectional shifts across timepoints.

Ki67 demonstrated substantial within-patient remodeling: Ki67 decreased in 10/18 cases (55.6%), increased in 4/18 cases (22.2%), and remained unchanged in 4/18 cases (22.2%), supporting dynamic proliferative modulation under therapy and/or sampling effects.

#### 3.4.1. Hormone Receptor Conversion

Across the paired-tissue subset, estrogen receptor (ER) status was fully concordant between core needle biopsy and surgical resection specimens, with no cases of ER loss or gain (0/18; 0.0%). Both triple-negative tumors with paired data (cases 9 and 25) remained ER−/PR− at surgery, indicating phenotypic stability of hormone receptor negativity. In contrast, PR status demonstrated clinically relevant conversion. Among ER-positive paired tumors (16/18; 88.9%), PR loss (ER+PR+ to ER+PR−) occurred in 6 of 16 cases (37.5%), corresponding to 6 of 18 (33.3%) of all paired cases; no PR gain was observed. PR loss showed subtype enrichment, occurring most frequently in Luminal B tumors (3/8; 37.5%) and Luminal B HER2+ tumors (2/2; 100%), with an additional event in Luminal A disease (1/6; 16.7%). These findings indicate that PR conversion was not restricted exclusively to the highest-proliferation luminal category. Within this cohort, PR loss emerged as the dominant pattern of hormone receptor discordance, whereas ER status remained stable. Given the established association between PR loss and diminished endocrine sensitivity, the observed rate of PR conversion reflects biologically and clinically meaningful molecular drift between diagnostic biopsy and surgical resection.

#### 3.4.2. HER2 Variability

Within the paired tissue subset, the HER2 IHC phenotype was not fully stable between the diagnostic core biopsy and the surgical specimen. A change in HER2 score (0/1+/2+/3+) was observed in 6 cases (33.3%), indicating that approximately one-third of evaluable tumors exhibited measurable HER2 score drift across timepoints and/or tissue compartments. The observed transitions included both upward and downward shifts. Notably, one case demonstrating an IHC upward shift from 2+ to 3+ occurred concurrently with PR loss, suggesting coordinated phenotypic drift involving both hormone receptor signaling and HER2-associated biology within the same tumor. The frequency and bidirectionality of HER2 score shifts observed here support the view that HER2 is not uniformly stable across sampling timepoints, reinforcing the clinical rationale for reassessment when treatment decisions depend on current HER2 status, particularly in modern therapeutic contexts where relatively small score changes (HER2 low/ultralow) may alter eligibility for HER2-directed, or HER2-low targeted therapies.

Sensitivity analysis: Because score-level HER2 variability may not always translate into changes in treatment eligibility, we performed a sensitivity analysis focusing on clinically relevant HER2-positive boundary crossing (3+ vs. non–3+). In the paired cohort, only 2/18 tumors (11.1%) crossed the HER2-positive boundary: one converted from HER2-positive to non-HER2-positive (3+→2+), and one converted from non-HER2-positive to HER2-positive (2+→3+). This indicates that while HER2 IHC score discordance occurred in one-third of paired cases, clinically actionable category conversion was less frequent.

#### 3.4.3. Ki67 Variability

Assessment of proliferative activity by Ki67 demonstrated substantial variability between diagnostic core biopsy and surgical resection specimens. Ki67 decreased in 10 of 18 cases (55.6%), increased in 4 of 18 (22.2%), and remained unchanged in 4 of 18 (22.2%), indicating dynamic modulation in 14 of 18 tumors (77.8%). The median change was −5 percentage points (IQR −13.8 to 0.0), with a range of −55 to +20 percentage points, reflecting a predominance of decreases, alongside a clinically relevant minority with increased proliferative activity.

Increases in Ki67 were observed across multiple baseline luminal categories, including Luminal A and Luminal B tumors, indicating that proliferative drift was not confined to a single baseline subtype. The largest increase (+20 percentage points; case 2) occurred concurrently with PR loss, illustrating coordinated remodeling of proliferation and hormone receptor signaling within the same tumor over time.

**Sensitivity analysis:** To reduce susceptibility to inter-observer variability and minor sampling-related fluctuations, Ki67 change was additionally summarized using a ≥10 percentage-point threshold. Using this conservative definition, 6/18 paired tumors (33.3%) showed a ≥10-point decrease in Ki67, whereas 2/18 tumors (11.1%) showed a ≥10-point increase in Ki67. This supports that a substantial subset of tumors exhibited suppressed proliferation, while marked increases in proliferation were uncommon.

#### 3.4.4. Molecular Subtypes—Luminal a to Functional Luminal B: Proliferation-Driven Drift

In our cohort, transition from Luminal A to a functional Luminal B phenotype was infrequent and characterized primarily by changes in proliferative activity rather than loss of estrogen receptor (ER) expression. These findings are consistent with the concept that a subset of ER-positive tumors may shift toward a higher-risk luminal state through proliferative remodeling without overt ER destabilization. Among initial Luminal A tumors (11/32; 34.4%), paired surgical receptor and Ki67 data were available for 6 of 11 cases (54.5%). Within this evaluable subset, Ki67 increased in 1 of 6 cases (16.7%), rising from 10% at diagnosis to 30% at surgery, consistent with functional luminal risk escalation. PR loss occurred in 1 of 6 cases (16.7%). Notably, this change was accompanied by a reduction in Ki67 (15% to 5%). In the Luminal A tumor, concurrent PR loss and Ki67 increase were observed within the same paired comparison (0/6).

Pre-treatment tissue next-generation sequencing revealed frequent PI3K pathway alterations within Luminal A tumors, with pathogenic PIK3CA variants identified in 4 of 11 cases (36.4%) and AKT pathway variants in 1 of 11 cases (9.1%). These findings are biologically consistent with pre-existing PI3K/AKT signaling in luminal tumors, which may attenuate ER dependence and contribute to reduced endocrine sensitivity without immediate phenotypic reclassification.

#### 3.4.5. HER2-Positive/HER2-Enriched Tumors: A High-Proliferation, Instability-Associated Phenotype

**Figure 2:**
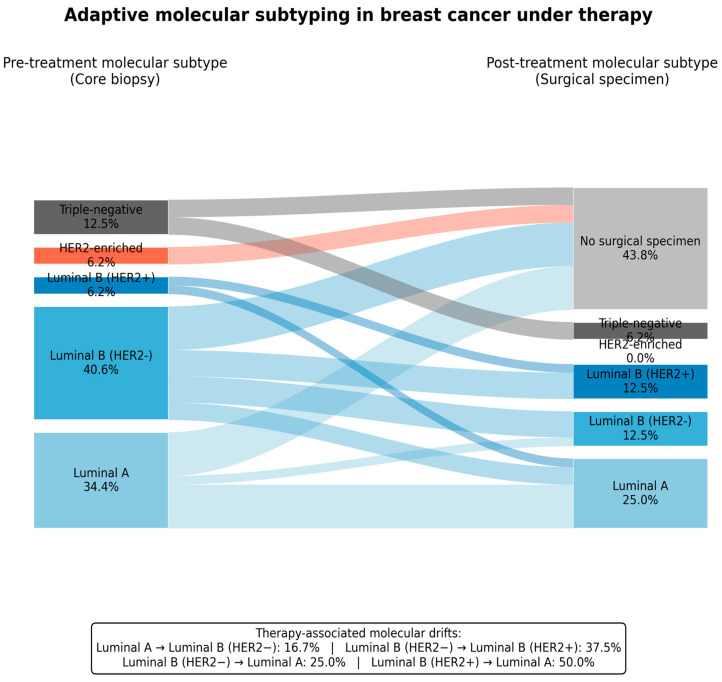
Adaptive molecular subtyping in breast cancer under therapy. Sankey diagram shows molecular subtype transitions from pre-treatment core biopsy to post-treatment surgical specimens. Node widths indicate patient proportions and flows represent subtype changes under therapy. Percentages are calculated across the cohort, with cases lacking post-therapy tissue shown separately. The inset summarizes therapy-associated molecular drifts, expressed as the proportion of tumors within each baseline subtype that changed subtype after treatment.

HER2-driven categories constitute a small but biologically distinct subset of the cohort, comprising HER2-enriched tumors (2/32; 6.3%) and Luminal B HER2-positive tumors (2/32; 6.3%). Despite the small sample size, these cases exhibited molecular and phenotypic features consistent with an aggressive, genomically unstable tumor. Pre-treatment tissue next-generation sequencing identified a TP53 pathogenic variant in 1 of 2 HER2-enriched tumors (50.0%; case 31), whereas TP53 alterations were not detected in Luminal A or Luminal B (HER2-negative) tumors. Although exploratory, this distribution aligns with the well-established enrichment of TP53 alterations in aggressive breast cancer subtypes and supports an instability-associated evolutionary trajectory in HER2-driven disease. Consistent with this genomic context, HER2-enriched tumors demonstrated high baseline proliferative activity, with Ki67 values reaching up to 80% in one case. The convergence of high proliferative activity and TP53 alteration supports a biologically plausible model in which HER2-enriched tumors exhibit an intrinsically unstable genomic substrate, facilitating early clonal diversification and adaptive evolution under therapeutic pressure. This interpretation is supported by recent molecular profiling studies linking the HER2-enriched subtype to high proliferative signaling and TP53-associated genomic instability [32] (Figure 2).

### 3.5. PI3K/AKT Pathway Alterations and Endocrine Resistance Biology

In this study, pre-treatment alterations affecting the PI3K/AKT pathway were identified in a clinically relevant subset of tumors. Pathogenic variants in PIK3CA were detected in 7 of 32 cases (21.9%), AKT pathogenic variants in 2 of 32 cases (6.3%), and combined PIK3CA and/or AKT alterations in 9 of 32 tumors overall (28.1%). These alterations were not evenly distributed across subtypes but clustered predominantly within luminal disease. Nearly half of Luminal A tumors (5/11; 45.5%) harbored a PIK3CA and/or AKT alteration, compared with 3 of 13 Luminal B (HER2-negative) tumors (23.1%). No PI3K/AKT pathway alterations were identified in TNBC (0/4). Among HER2-enriched tumors, 1 of 2 cases (50.0%) carried a PIK3CA pathogenic variant. Although the numbers are limited, this pattern underlies the preferential association of PI3K/AKT pathway dysregulation with ER-driven breast cancer biology. This subtype-specific distribution is biologically coherent with the established role of the PI3K/AKT/mTOR pathway as a dominant regulator of cell survival and growth in ER-positive BC. From a biological standpoint, activation of the PI3K/AKT pathway maintains proliferative and survival signaling independently of estrogen receptormediated transcription, enabling continued tumor growth despite pharmacologic suppression of ER signaling. Clinically, this biology has translated into biomarker-guided therapeutic strategies in advanced HR^+^/HER2^−^ disease. In particular, randomized data from CAPItello-291 demonstrated improved progression-free survival with capivasertib plus fulvestrant in both the overall population and in tumors harboring PIK3CA/AKT1/PTEN alterations, leading to regulatory approval and guideline adoption [26,33].

#### 3.5.1. FGFR Amplification

In the present cohort, FGFR1 amplification was identified in a single case (1/32; 3.1%) and was detected exclusively through plasma ctDNA analysis. Although infrequent, this finding is clinically informative. Detection of FGFR1 amplification is clinically important because FGFR pathway activation identifies estrogen receptor-positive tumors with an increased propensity for resistance to endocrine therapy and CDK4/6 inhibition, reflecting reliance on alternative growth pathways when ER-driven control is therapeutically suppressed [34].

#### 3.5.2. TP53 Alterations and High-Risk Biological States

TP53-related alterations were infrequent in this cohort but biologically informative. A TP53 pathogenic variant (PV) was detected in baseline tumor tissue in 1/32 cases (3.1%), occurring in a HER2-enriched tumor (case 31). Although this baseline frequency appears lower than expected from large breast cancer datasets, it reflects the small cohort size, luminal predominance, and the use of strict pathogenicity-based reporting thresholds.

It should be noted that TP53 alterations detected in this study include pathogenic variants (PV), variants of uncertain significance (VUS), and unclassified plasma-only variants (UPV). Only variants meeting pathogenic classification criteria were included in the reported TP53 PV frequency. Additional TP53 signals observed in plasma ctDNA or classified as VUS/UPV likely reflect subclonal events, low-allele-fraction alterations, or technical classification limitations rather than definitive truncal driver mutations. Germline TP53 alterations were not assessed in this study.

**Figure 3:**
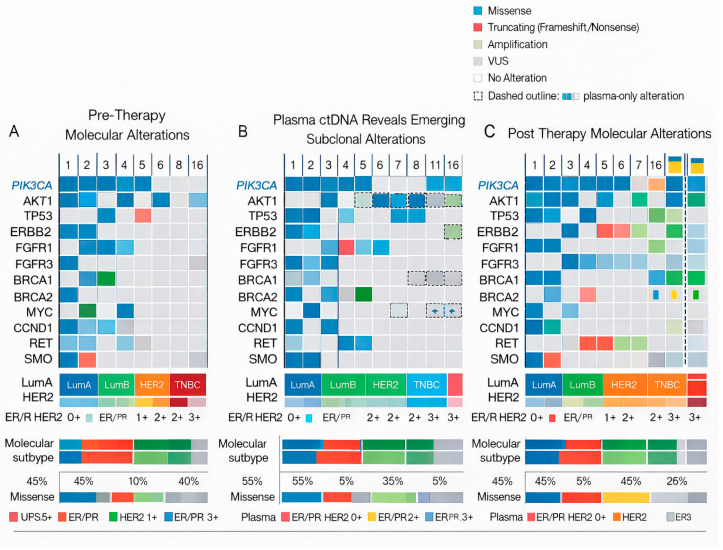
Longitudinal molecular alterations across tissue and plasma in breast cancer. Oncoprint-style heatmaps show genomic alterations across three disease stages: (**A**) pre-therapy diagnostic biopsies, (**B**) plasma ctDNA-derived subclonal alterations, and (**C**) post-therapy surgical specimens. Rows represent recurrently altered genes and columns individual patients. Alterations are color-coded by variant class (missense, truncating [frameshift/nonsense], amplification, or variant of uncertain significance), with white indicating no alteration; dashed outlines denote alterations detected only in plasma ctDNA. Annotation tracks show intrinsic subtype and hormone receptor/HER2 status at each time point. Bar plots summarize alteration type distributions. Overall, the figure highlights clonal persistence, therapy-associated subclonal events, and dynamic molecular evolution. TP53 alterations shown in this figure include pathogenic variants (PV), variants of uncertain significance (VUS), and unclassified plasma-only variants (UPV). Only one TP53 alteration met pathogenic criteria in core biopsy diagnostic tissue sequencing; additional TP53 signals represent low-confidence or plasma-detected subclonal alterations and are displayed to illustrate longitudinal molecular heterogeneity rather than pathogenic mutation frequency.

Figure 3 summarizes TP53 calls across compartments and timepoints, including plasma ctDNA, where a TP53 VUS was detected in a TNBC case, supporting the concept that subclonal alterations may be detectable outside the diagnostic biopsy sample. A TP53 pathogenic variant was identified in baseline tissue in 1 of 32 cases (3.1%), occurring in a HER2-enriched tumor with concurrent HER2 amplification (case 31). In plasma, a TP53 variant of uncertain significance (VUS) was detected in 1 of 32 cases (3.1%), observed in a triple-negative breast cancer (TNBC) case. Although pathogenic variants and VUS differ in their clinical actionability, both findings arose in biologically aggressive disease contexts. Consistent with the literature, TP53 alterations are strongly associated with genomic instability, adverse clinical outcomes, and enrichment in high-risk breast cancer subtypes, including HER2-positive and triple-negative disease. In the present cohort, detection of a TP53 alteration in HER2-enriched disease supports a model of increased evolutionary capacity, in which genomic instability may facilitate clonal diversification and enhance resistance potential under therapeutic pressure [35].

#### 3.5.3. Biological Interpretation of the Low TP53 Rate

We consider that the low frequency of pathogenic TP53 mutations observed in this cohort (1/32; 3.1%) is both biologically and methodologically consistent with the characteristics of the studied population. The cohort is predominantly composed of hormone receptor-positive, luminal breast cancers, a molecular context in which TP53 alterations are substantially less frequent than in triple-negative or HER2-enriched disease. Approximately 78% of patients were classified as luminal subtypes (Luminal A, Luminal B, and Luminal B HER2+). Accordingly, no TP53 pathogenic variants were detected in luminal tumors, whereas the single TP53 pathogenic alteration occurred in a HER2-enriched case characterized by high proliferative activity, consistent with an instability-associated biological phenotype.

It is well established in the literature that the frequency of TP53 mutations varies substantially across intrinsic molecular subtypes of breast cancer and is significantly lower in ER-positive/luminal tumors compared to ER-negative subtypes such as triple-negative breast cancer (TNBC) and HER2-enriched disease. Large genomic studies, including the METABRIC dataset, have consistently demonstrated this pattern. In METABRIC, TP53 mutations are relatively uncommon in luminal tumors, with reported frequencies of approximately 5% in Luminal A and ~13% in Luminal B breast cancers [36], in contrast to much higher rates in basal-like/TNBC and HER2-enriched subtypes.

**Figure 4:**
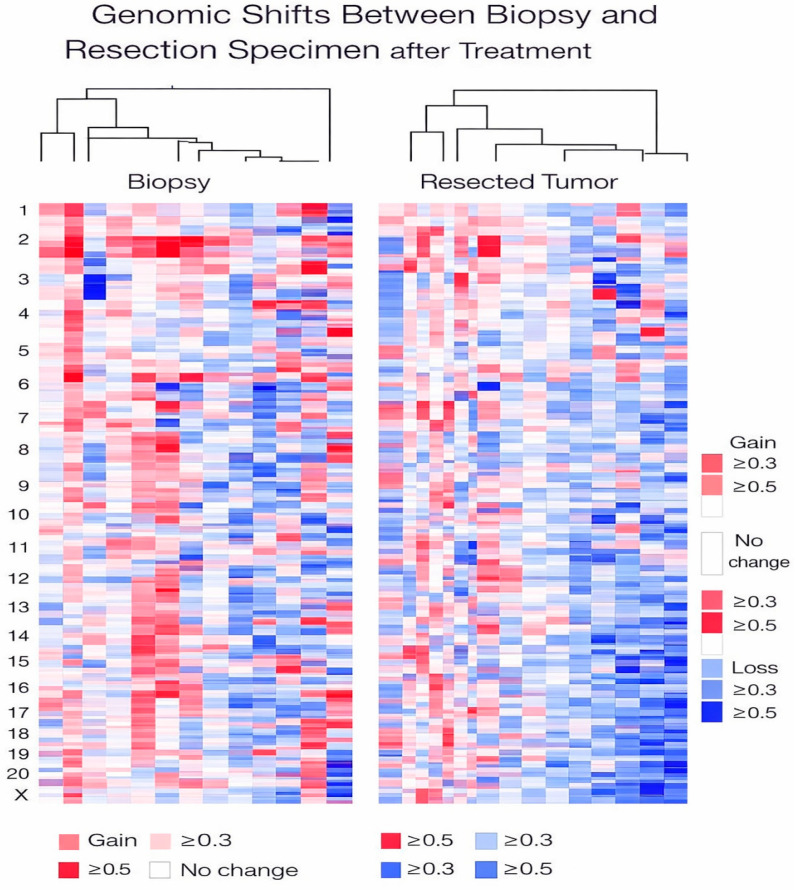
Genomic copy-number alterations in paired core biopsy and resection specimens. Heatmap showing genome-wide copy-number alterations detected by next-generation sequencing (Ion Torrent platform, Oncomine Comprehensive Assay Plus) in paired tumor samples obtained at diagnosis (core biopsy, (**left**)) and after treatment (surgical resection, (**right**)). Rows represent chromosomes 1–22 and X, and columns correspond to individual patients. Copy-number gains are depicted in red and copy-number losses in blue, with light shading indicating alterations ≥0.3 and dark shading indicating alterations ≥0.5. White denotes no detectable copy-number change. Hierarchical clustering was performed separately for biopsy and resection specimens to visualize similarities and divergences in genomic profiles, illustrating treatment-associated genomic remodeling and intratumoral heterogeneity between baseline and post-therapy tumor compartments.

Importantly, although pathogenic TP53 events were rare in our cohort, genome-wide copy-number analysis revealed evidence of broader genomic remodeling between baseline and post-treatment specimens. Hierarchical clustering of paired core biopsy and surgical samples demonstrated divergence in genome-wide copy-number profiles, illustrating therapy-associated genomic remodeling and intratumoral heterogeneity (Figure 4).

Given the predominance of ER-positive/luminal cases in our cohort, a lower overall frequency of TP53 pathogenic variants would therefore be expected based on these published subtype-specific mutation rates. While our reported pathogenic TP53 mutation rate in tissue NGS is 3.1% (1/32), this should be interpreted in the context of both the subtype distribution of our cohort and the conservative variant classification criteria used. When including all TP53 calls (pathogenic, VUS, and unclassified plasma-only variants), the frequency increases to 6.3% (2/32), further aligning with expectations for a predominantly luminal cohort.

In this context, the low TP53 pathogenic mutation rate should not be interpreted as an absence of molecular evolution. Rather, it suggests that in this luminal-dominant cohort, tumor adaptation under therapeutic pressure is primarily mediated through functional signaling reprogramming—such as PI3K/AKT pathway activation, progesterone receptor loss, and proliferative remodeling—rather than through widespread genomic instability driven by TP53 inactivation. TP53 alterations in this study therefore appear restricted to biologically aggressive contexts, indicating that TP53-driven evolution is mainly confined to more aggressive subtypes [37].

#### 3.5.4. Mammographic Density as a Potential Modifier of TP53 Mutation Frequency

Beyond biological heterogeneity discussed above, an additional factor that may influence the frequency of TP53 mutations in breast cancer cohorts is mammographic density (MD). Emerging evidence suggests that MD is associated not only with breast cancer risk but also with distinct tumor biology and tumor–stroma interactions that could shape both the true prevalence and detectability of TP53 alterations. Recent comparative analyses have reported a markedly lower frequency of TP53 mutations in cancers arising in women with high mammographic density compared with those in low-density breasts, raising the possibility that dense breast tissue may provide a pro-tumorigenic stromal and immune microenvironment that reduces the selective pressure for TP53 inactivation during tumor evolution [38,39]. Although tumors from high (HMD) and low (LMD) mammographic density breasts were largely comparable in their driver gene landscapes and copy number profiles, a key divergence was observed in TP53 mutation frequency, which was significantly higher in cancers arising in LMD breasts and relatively depleted in HMD-associated tumors. This suggests that breast cancers developing in LMD tissue may rely more heavily on intrinsic genomic disruption—particularly TP53 loss—for malignant progression, whereas cancers in HMD tissue may be driven to a greater extent by extrinsic, microenvironmental influences within the dense breast stroma.

Whole-genome sequencing further revealed density-associated differences in underlying mutational processes: HMD tumors demonstrated increased contribution of the SBS3 homologous recombination deficiency (HRD) signature and the SBS1 aging/deamination signature, whereas LMD tumors exhibited higher levels of APOBEC-associated mutagenesis (SBS2/13). These findings indicate that genomic instability in HMD tumors may arise through mechanisms distinct from TP53 disruption. Complementing these genomic analyses, tumor-infiltrating lymphocyte (TIL) assessment showed that HMD tumors harbor a more immune-active microenvironment, with significantly greater infiltration of CD8+ T cells, CD4+ T cells, and B lymphocytes within the tumor epithelium, as well as higher overall stromal TILs. Therefore, breast cancers arising in low-density tissue are more genomically driven (with higher TP53 mutation rates and APOBEC activity), whereas cancers arising in high-density tissue are more microenvironment-driven, characterized by HRD-associated genomic instability and a tumor-promoting immune landscape. These insights provide a potential biological explanation for the poorer clinical outcomes and higher interval cancer rates observed in women with high mammographic density, and suggest that density-specific therapeutic strategies—particularly those targeting immune or DNA repair pathways—may be warranted

Conversely, several methodological factors linked to MD could artifactually lower the apparent TP53 mutation rate in high-density cases. Tumors arising in dense breasts tend to have higher stromal content and lower tumor purity. Lower tumor purity proportionally reduces the effective coverage of variant alleles from cancer cells, thereby lowering variant allele fractions (VAFs) and reducing the sensitivity of mutation detection with standard next-generation sequencing thresholds [40,41,42]. In clinical specimens such as small biopsies, where tumor cellularity may be low, this reduction in effective allele fraction can make true somatic mutations harder to detect and increase the likelihood of false-negative results.

#### 3.5.5. Mammographic Density Distribution by ACR Category and Molecular Subtype

**Figure 5:**
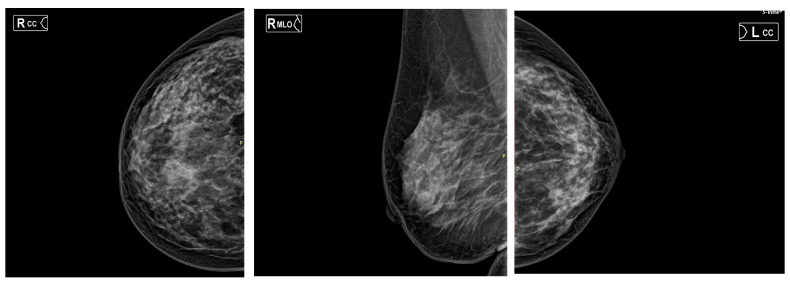
Mammographic image demonstrating predominantly dense fibroglandular tissue with minimal fatty background, consistent with extremely dense breast parenchyma (ACR category D).

Mammographic density was systematically quantified in the present study. Based on mammographic density findings (ACR A–D), the cohort (n = 32) was predominantly characterized by high breast density) [43]. Overall, 78.1% of patients were classified as ACR C or D, including 40.6% with extremely dense breasts (ACR D) and 37.5% with heterogeneously dense breasts (ACR C), whereas only 21.8% were low-density breasts (ACR A or B). Distribution of estimated density also varied by molecular subtype (Figure 5). All triple-negative breast cancers (TNBC) were classified as ACR D (100%), suggesting a strong association between this aggressive subtype and very high mammographic density. In Luminal B tumors, 77.0% were estimated as ACR C/D, reflecting a relationship between higher proliferative activity and increased density. In comparison, Luminal A tumors showed a more balanced distribution, with 36.4% in ACR A/B and 63.6% in ACR C/D. HER2-enriched tumors (n = 2) were evenly split between ACR C and ACR D (Table 2). 

## 4. Discussion

From static classification to adaptive molecular subtypes in breast cancer. The present study supports a conceptual shift from static, diagnosis-based molecular classification toward a dynamic, adaptive model of breast cancer biology. By integrating paired tissue- and plasma-based molecular profiling obtained before and after treatment, our findings indicate that breast cancer molecular phenotypes are not fixed entities but evolve over time in response to therapeutic pressure and microenvironmental influences. Within this dynamic framework, discrepancies observed between different sampling modalities are increasingly recognized as biologically informative rather than merely technical limitations. Prior studies have consistently demonstrated that molecular concordance across core biopsy, surgical resection specimens, and plasma-derived circulating tumor DNA (ctDNA) in breast cancer is influenced by tumoral subclones, therapeutic pressure, specimen type, and overall tumor burden. Importantly, that discordance among these sampling modalities is not solely a technical limitation but rather a biological consequence of intratumoral heterogeneity and ongoing clonal evolution [44]. Breast cancers are markedly heterogeneous and evolve through branching clonal dynamics, in which early clonal (“truncal”) driver alterations are shared across tumor regions, whereas later subclonal (“branch”) events are spatially restricted and shaped by microenvironmental influences and therapeutic selection. As a result, core needle biopsies provide a limited representation of the tumor genome, and even surgical resection specimens reflect an aggregate of heterogeneous subclonal populations rather than a single, uniform genomic profile. Consequently, truncal driver alterations tend to show high concordance across biopsy, resection, and plasma specimens, while subclonal events represent the principal source of molecular discordance across sampling modalities, reflecting ongoing clonal evolution under therapeutic pressure.

### 4.1. Molecular Plasticity Under Therapeutic Pressure

A central observation of this cohort is the high frequency of post-treatment phenotypic and molecular drift, particularly within HR-positive disease. While ER expression remained stable across paired specimens, PR loss occurred in approximately one-third of evaluable cases, predominantly within luminal B and luminal B HER2-positive tumors. This selective receptor conversion pattern is biologically coherent and consistent with prior evidence that PR loss reflects functional disruption of estrogen signaling rather than complete ER pathway abrogation. Notably, PR is increasingly recognized as more biologically labeled than ER and is widely regarded as a downstream marker of functional ER signaling. Accordingly, loss of PR expression in ER-positive breast cancer has been consistently associated in recent systematic reviews and meta-analyses with diminished responsiveness to endocrine therapy and less favorable clinical outcomes [44]. Such changes are increasingly recognized as early indicators of endocrine resistance and may precede overt clinical progression.

Similarly, variability in HER2 immunohistochemical scores and marked Ki67 variation highlight the instability of key biomarkers used for subtype allocation and treatment selection. HER2 discordance between core needle biopsy and surgical specimens is well documented, with particular attention in recent work to instability across the HER2-ultra-low/HER2-low boundary and the clinical consequences of score-level shifts. Post-treatment conversion has also been reported after neoadjuvant therapy, including both positive → negative and negative → positive changes.

Nearly 80% of paired tumors demonstrated Ki67 changes, with both suppressive and paradoxical proliferative responses observed. The predominance of Ki67 decreases is compatible with treatment-associated suppression of proliferation and/or differences in sampled tumor regions. Importantly, proliferative increases were not restricted to high-risk baseline subtypes, indicating that functional luminal risk can escalate even in tumors initially classified as luminal A. These findings reinforce the limitations of static subtype assignment and emphasize that therapy-induced biological remodeling may substantially alter tumor behavior beyond what baseline classification predicts.

Across molecular subtypes, longitudinal profiling revealed distinct yet convergent patterns of functional evolution. Luminal A tumors commonly harbored baseline PI3K/AKT pathway alterations, with a minority showing drift toward increased proliferative signaling over time. Luminal B tumors demonstrated greater evolutionary plasticity, with some cases evolving toward HER2 pathway dominance, as evidenced by HER2 escalation accompanied by PR loss. Although limited in number, HER2-enriched tumors exhibited consistently high proliferation and genomic instability, reflecting intrinsically aggressive biology. Collectively, these findings show the value of longitudinal, multi-compartment profiling in capturing clinically relevant subtype evolution beyond what can be inferred from a single diagnostic biopsy.

### 4.2. Adaptive Molecular Subtyping as a Clinically Relevant Framework

Our observations align with emerging evidence from large neoadjuvant and translational studies demonstrating that adaptive molecular subtypes, defined by therapy-induced transcriptional and phenotypic changes, outperform intrinsic subtypes in prognostic stratification. In this context, molecular subtype transitions in our cohort were primarily driven by changes in proliferation and receptor signaling rather than complete lineage switching, supporting a model of functional subtype plasticity. This adaptive perspective has direct clinical implications. Tumors demonstrating favorable molecular adaptation (e.g., Ki67 suppression without receptor destabilization) may have a substantially better prognosis than suggested by residual disease burden alone, whereas tumors exhibiting PR loss, rising Ki67, or emergent pathway activation may warrant treatment intensification or molecularly guided escalation strategies.

### 4.3. Genomic Drivers of Adaptation and Resistance

At the genomic level, alterations affecting the PI3K/AKT signaling axis emerged as a dominant feature of luminal tumors, with nearly one-third of cases harboring PIK3CA and/or AKT pathogenic variants. This enrichment within luminal A disease suggests that pathway activation may represent an early adaptive state, enabling partial estrogen independence without immediate phenotypic conversion. Such biology provides a plausible explanation for attenuated endocrine sensitivity and supports contemporary strategies combining endocrine therapy with PI3K or AKT pathway inhibition in selected patients. Conversely, alterations associated with genomic instability, including TP53 mutations and MYC alterations, were rare but confined to biologically aggressive contexts such as HER2-enriched and triple-negative disease.

TP53 is among the most frequently altered genes in breast cancer, with reported somatic mutation rates varying substantially by intrinsic subtype and clinical context. In particular, TP53 alterations are enriched in triple-negative and HER2-enriched tumors, whereas luminal tumors show lower prevalence. In our cohort, the low baseline TP53 PV detection rate should therefore be interpreted cautiously, given the modest sample size, subtype distribution, sampling constraints, and the fact that ctDNA results frequently yielded UPV/VUS classifications rather than definitive pathogenic calls.

This distribution supports a model in which genomic instability facilitates rapid clonal diversification and resistance under treatment pressure, particularly in HER2-driven tumors. Although limited by sample size, these patterns are concordant with large genomic datasets and reinforce subtype-specific evolutionary trajectories.

### 4.4. Complementary Value of Liquid Biopsy in Longitudinal Assessment

Plasma ctDNA analysis provided non-redundant molecular information in over half of the cohort and identified clinically relevant alterations not captured by tissue profiling, including FGFR1 amplification and BRCA1/2 variants. These findings demonstrate the utility of liquid biopsy as a dynamic surveillance tool that captures spatial heterogeneity and emergent resistance mechanisms. While many plasma findings were classified as variants of uncertain pathogenicity, their occurrence within biologically aggressive or evolving disease contexts highlights the importance of interpretive frameworks that integrate ctDNA results with phenotypic, temporal, and clinical data rather than relying on isolated variant calls. From a translational standpoint, even a modest rate of plasma-only actionable findings is meaningful within an adaptive care model, as these represent scenarios in which tissue-only strategies would delay or miss identification of targetable biology. Our data support the use of ctDNA as a complementary component of longitudinal molecular assessment rather than a replacement for tissue-based evaluation.

### 4.5. Addressing Statistical and Analytical Limitations

We acknowledge that retrospective longitudinal biomarker studies can be vulnerable to multiplicity, underpowered subgroup comparisons, and descriptive overinterpretation of subtype instability. To strengthen interpretability, we predefined a limited set of primary paired endpoints (PR conversion, HER2 score discordance, and paired Ki67 change) and applied appropriate paired null-hypothesis tests for categorical outcomes. Specifically, PR conversion demonstrated a directional loss-only pattern (McNemar exact *p* = 0.031), whereas HER2 score discordance was bidirectional and consistent with symmetric redistribution (Bowker *p* = 0.57). We further implemented sensitivity analyses designed to emphasize clinically meaningful and robust changes, including HER2-positive boundary crossing (11.1%) and Ki67 ≥ 10-point thresholds. Together, these refinements enhance the robustness of the core findings while appropriately limiting inferences about causality, treatment-specific effects, and prognostic impact, which require larger prospective cohorts with standardized sampling and treatment annotation.

## 5. Conclusions

From our perspective, molecular adaptive subtyping reframes breast cancer classification as a longitudinal, evolution-aware process. We believe that the emerging standpoint is complementarity: paired tissue + ctDNA profiling (and, where feasible, longitudinal ctDNA) increases the probability of identifying clinically relevant alterations and tracking resistance evolution, rather than expecting perfect cross-compartment concordance. Cross-specimen molecular shifts reflect the complex interplay between tumor clonal architecture (truncal vs. branch), spatial sampling (biopsy vs. resection specimen), tumor burden, and temporal dynamics under therapy (selection and residual burden), rather than a simple function of assay performance alone. While limited by cohort size and incomplete longitudinal sampling, observed patterns mirror biological mechanisms repeatedly reported in larger neoadjuvant and metastatic studies, supporting their broader biological relevance. Future clinical trials, biomarker guidelines, and therapeutic algorithms should move beyond fixed intrinsic labels and be explicitly designed around evolving molecular states—aligning precision oncology with the dynamic nature of breast cancer biology.

## Figures and Tables

**Table 1:** Longitudinal biomarker concordance and discordance between diagnostic core biopsy and post-treatment surgical specimens in paired breast cancer cases (n = 18), including estrogen receptor (ER), progesterone receptor (PR), HER2 immunohistochemistry (IHC) score, and Ki67 proliferation index, with corresponding agreement statistics and paired-test results.

Biomarker	Paired Cases (n)	Concordant Cases n (%)	Discordant Cases n (%)	Direction of Change	Agreement Statistic	*p*-Value
Estrogen receptor (ER)	18	18 (100%)	0 (0%)	No gain or loss observed	Cohen’s κ = 1.00	Not applicable
Progesterone receptor (PR)	18	12 (66.7%)	6 (33.3%)	PR loss only (ER^+^PR^+^ → ER^+^PR^−^)	Cohen’s κ = 0.44 (moderate)	McNemar *p* = 0.031
HER2 IHC score (0–3+)	18	12 (66.7%)	6 (33.3%)	Both upward and downward shifts	Weighted κ = 0.52	Not tested *
Ki67 (%)	18	4 (22.2%)	14 (77.8%)	↓ in 10 (55.6%); ↑ in 4 (22.2%)	—	Wilcoxon *p* = 0.018

**Table 2:** Table summarizes the distribution of mammographic density categories (ACR A–D) across the cohort (n = 32). The majority of patients were classified as having high breast density, with 40.6% in ACR category D (extremely dense) and 37.5% in ACR category C (heterogeneously dense). In contrast, only 21.8% of cases were categorized as low-density breasts (ACR A or B), including 15.6% with almost entirely fatty breasts (ACR A) and 6.2% with scattered fibroglandular density (ACR B).

ACR Category	Number of Cases	% of Cohort
A (almost entirely fatty)	5	15.6%
B (scattered fibroglandular density)	2	6.2%
C (heterogeneously dense)	12	37.5%
D (extremely dense)	13	40.6%

## Data Availability

https://scienceonlinero.wixsite.com/research/66pte-oncoguard (accessed on 8 February 2026).
